# Chemical restraint as behavioural euthanasia: case studies from the Royal Commission into Aged Care Quality and Safety

**DOI:** 10.1186/s12877-023-04116-5

**Published:** 2023-07-19

**Authors:** Patricia Cain, Pelden Chejor, Davina Porock

**Affiliations:** Centre for Research in Aged Care, School of Nursing and Midwifery, Edith Cowan University, 270 Joondalup Drive, 6027 Joondalup, WA Australia

**Keywords:** Chemical restraint, Aged care, Dementia, Royal Commission, Case study, Psychotopic medication, Family advocacy

## Abstract

**Background:**

The prescription of psychotropic medication to older people living with dementia in residential aged care has become an increasing concern. The use of prescription medication is often prefaced as a way of preventing harm to self and others. However, the use of such medications has been considered a way of managing some of the behavioural and psychological symptoms of dementia. Using a large secondary data set, this study aimed to identify the precursors and mediating factors that influence the use of chemical restraint of older people in residential aged care.

**Methods:**

Publicly available documents from the Australian Royal Commission into Aged Care Quality and Safety were used as the data corpus for this study. Keywords were used to search over 7000 documents to extract a set of topic-related content. We identified the cases of seven people in respite or permanent residential aged care who had been prescribed or administered psychotropic medication under circumstances that appeared to demonstrate chemical restraint. All documents relating to the cases were collated for our data set. A descriptive case study approach to analysis was taken.

**Results:**

Four key descriptive patterns were identified: labelling and limits to tolerance, pushing prescription as a solution, coverups and avoiding consent, and family’s fight for liberty. Triangulation across the data and academic literature supports the findings.

**Conclusion:**

Our findings provide some insight into how chemical restrain happens. Featuring throughout the cases were reports of a lack of workforce capacity to care for and support residents exhibiting dementia behaviours. Prescription of psychotropic medications featured as a “first resort” care solution. Family and friends found such approaches to care unacceptable and frequently challenged the practice. Where consent for prescription was explicitly denied, more covert approaches are demonstrated. Family awareness, presence, and advocacy were key to challenging the practice of chemical restraint. Shortfalls in the capacity of the current workforce come into play here. However, workforce shortcomings can no longer mask this ubiquitous practice. Just as importantly the spotlight needs to be turned on the prescribers and the providers.

## Background

Following a series of aged care reviews [1] and investigative journalism exposés of substandard and abusive care in Australian residential aged care, in 2018, the Governor-General established a Royal Commission into Aged Care Quality and Safety (Henceforth Royal Commission). The public, including consumers of aged care, their families, carers, aged care workers, providers, and health professionals, were encouraged to make submissions, and over 10,000 submissions were received. Following Royal Commission guidelines, submissions and evidence of the proceedings have been made publicly available via the Royal Commission website [2]. In October 2019, the Royal Commission released a lengthy and damning three-volume Interim Report titled *‘Neglect’* [3]. One of the issues spotlighted in the Interim Report was the use of chemical restraint to manage behaviours, particularly of people with dementia living in residential care. The Interim Report stated: “Unfortunately, chemical and physical restraint is the easiest ‘care’ practice for many, but it is a pathway for people with dementia that is not in line with our human rights or best practice” [3]. The final report declared that urgent reforms were necessary to protect older people from the use of potentially harmful chemical restraint. However, further comments made in Commission documents permitted chemical restraint as part of an independently assessed behavioural support plan.

In Australia, the term “chemical restraint” refers to the inappropriate use of psychotropic medications such as antipsychotic, antianxiety, antidepressant, and sedative medications for the primary purpose of controlling or restricting a person’s behaviour or movement [4]. In contrast, the appropriate use of psychotropic medications for people living with dementia may occur where people have coexisting mental health conditions or distressing psychotic symptoms, or where people pose a threat to themselves or others. Australian practice guidelines recommend prescription should only be made following extensive consultation with the person living with dementia (or their substitute decision-maker), their family, the prescriber, and direct care providers. Additionally, guidelines stipulate that prescription should be regularly reviewed, and non-pharmacological strategies maintained [5]. The inappropriate use of psychotropic medicines as a way of managing dementia behaviours that may be challenging for caregivers to work with [6] is relatively common practice in Australian residential care [7–9] as well as internationally [10–12]. A recent review on the prevalence of psychotropic medication use in Australian aged care facilities showed that 13 − 42% of people in residential care were prescribed psychotropics such as antipsychotic and sedative medications at rates considerably higher than would be expected for appropriate treatment purposes [13]. Indeed, The Australian Government’s Aged Care Clinical Advisory Panel estimated that only 10% of the psychotropic medications used in residential aged care were justified in the course of treatment for mental health conditions and some rare symptoms associated with dementia [3].

Multiple antecedents have been cited for the use of chemical restraint in residential aged care, including client factors surrounding communication difficulties [9, 14] behavioural and psychotic symptoms [10, 15–17] as well as workforce factors including low staff to patient ratios [17–19], knowledge and skills mix [17, 20], and workforce attitudes [21]. The use of restraint, both physical and chemical, is often premised on preventing harm to individuals and others [22, 23] and, reducing falls risk [24]. However, chemical restraint can result in decreased functioning and physical deconditioning which may lead to falls as well as cognitive decline, [25] and can cause respiratory depression [26]. The use of antipsychotic medication has also been indicated to increase the risk of stroke and cardiovascular events for people living with dementia [27, 28]. While something is known about the prevalence of chemical restraint, less is known about the more subtle precursors and mediating factors around the proposition of chemical restraint. Considering the attention already given to the topic, the publicly available evidence from the Royal Commission presented an opportunity to explore and describe in some detail the personal factors (both protective and risk) that influence the use of chemical restraint of older people in residential aged care.

The data used for this analysis is large-scale publicly available secondary qualitative data and presented a unique set of analytic challenges. In total, we had access to over 7,000 publicly available documents of various types from the Royal Commission web site; these included witness statements, hearing transcripts, and supporting evidence. Taking the scholar’s lens to the data *ex post facto*, and where there was no opportunity to dig deeper into the experience and complexity during interview or observation, Merriam’s definition of case study and pragmatist-constructivist approach was adopted [29]. Merriam’s epistemological stance assumes a reality that is both subjectively and socially constructed. Their approach to case study recognises a case as a bounded system of information upon which pragmatic analytic decisions, such as choice of quantitative or qualitative method, guide knowledge identification for progressing practical solutions. Our pragmatic-constructionist approach allows us to highlight the risk and protective factors that impact the use of chemical restraint at an individual level. For this investigation, a cross-case study approach using qualitative descriptive analysis emerged.

## Method

While the documents used in this study are freely available to the public, informants to the Royal Commission would not have anticipated their submissions and testimonies to be used for any other purpose, especially scholarly analysis. For this reason, we approached the Office of the Royal Commission and gained their approval to use the publicly available documents as data for our study. Due to many documents including overt identifiers and stories of vulnerable people, we also received full Human Research Ethics Council approval from our governing academic institution, including the waiver of consent requirement [30]. In reporting the data, as much as possible, we have maintained the contextual integrity of the evidence [31] and emulated the justice, beneficence, and respect afforded to all research participants. Guided by the work and recommendations of the Association of Internet Researchers [32], any identifying details have been kept to the minimum for descriptive purposes and we have omitted the names of people and institutions from extracts reported.

The documents used for this analysis comprise 227 verbatim transcripts of hearings where witnesses testified and were questioned by the Council Assisting the Commission and 5712 exhibits in the form of witness statements and documents supporting claims made at hearings. All data were downloaded into NVivo 12 [33], where we used keyword searches to identify relevant content [34]. Based on the literature and author experience, the research team developed an extensive list of 226 keywords relating to chemical restraint initiation, use, and outcomes. We used direct words such as chemical and restraint and names of psychotropic medications including antipsychotics, antidepressants, antianxiety, and sedative medicines, both generic and trade. We also searched for words associated with behavioural triggers for restraint, such as wander, shout, scream, aggressive, violent, hitting, biting, agitated, and anxious. Outcomes of the keyword searches allowed us to establish familiarity with the data and identify and isolate topic-relevant content. A large secondary data corpus requires a considered approach to enable content relevant to the research question to be identified. We drew on Davidson et al. [35] breadth and depth strategy which has been likened to stages of an archaeological survey. Our initial survey was broad; we examined the data for mentions of keywords across all document types. In reviewing the results, we noticed that particular people and stories began to reappear. It was here that we narrowed our focus and began looking in depth at specific cases to collate all case relevant data. This strategy revealed accounts of seven people who had been restrained (or restraint attempted) using psychotropic medications (see Table 1 for case details). All documents related to each person were captured to create our topic-specific dataset, and we took a cross-case study approach to analysis.

**Table 1:** Case summaries

Case	Age	Gender	Diagnostic information	Care type and length	Triggering incidents	Treatment outcomes	Treatment concerns	Presented or submitted evidence	Data type
1	< 70	Man	Lewy Body Dementia, Parkinson’s symptoms	Residential respite care and residential aged mental health care.	Identified as distressed, noncompliant, and aggressive during hallucinations.	Residents’ general health deteriorated, including weight loss. Reported by family as often drowsy and “groggy”.	Medications administered without consent. “problem” behaviours related to unsuitable medication and ineffective care and mislabelled as dementia symptoms	Spouse.	1 HT 1 WS
2	< 70	Man	Cognitive impairment	Two months residential respite care.	Identified as restless, wandering, and disruptive.	Resident left residential care in a deconditioned state, incontinent and with limited mobility and speech.	Medications administered without consent. Physical restraint also used on resident.	Spouse and adult child.	2 HT 3 PHT 8 WS 3 SE
3	n/a	Man	Cognitive impairment	Older persons mental health service. Two periods of residential care.	Identified as “too difficult to deal with”	Residents’ health rapidly declined. Injuries sustained in care suggest neglect and abuse.	Medicated for sedation with one instance of overmedication with antipsychotic medication. Unexplained bruising suggesting abuse.	Spouse and adult child.	1 HT 2 WS
4	< 80	Woman	Dementia	Living in residential care for 5 years.	Reported to be agitated and wandering.	On one occasion the resident was reported to be in a “semi-conscious state” for three days.	Medication administered without consent. Family noted that their mother was frequently falling asleep and difficult to rouse during visits. Agitation experienced by the resident was due to reliving childhood trauma.	Adult children, provider, community organisation, allied health professional, general practitioner.	1 HT 3 PHT 7 WS 1 SE
5	< 90	Woman	Macular degeneration	Entered low care residential aged care and moved to high level care.	Resident had frequent falls and was reported to call out and scream.	Resident lost a significant amount of weight and incurred a painful pressure ulcer which developed into a bone infection.	Medications administered without consent. Family observed their mother to be almost always sleepy and often unresponsive. Family believed pain inadequate pain management was the cause of the vocal behaviours.	Adult child, general practitioner, provider governance.	1 HT 4 PHS 1 WS 3 SE
6	< 80	Woman	Dementia	Living in residential care for 3 years	Reported agitation and aggression toward care givers.	Psychotropic medication not administered.	Medication prescribed without consent. Family members, including relatives with medical backgrounds, intervened to block administration. Pain and care worker approach were considered the cause of behaviours.	Adult child.	1 HT 1WS
7	< 90	Man	Dementia	Entered low care residential aged care and moved to high level care.	Reported to be resistant and wandering.	Psychotropic medication not administered.	Repeated requests to prescribe psychotropic medication were refused by family. Physical restraint also used on resident.	Adult children.	1 HT 1 WS

n/a = information not available

HT = Hearing transcript, PHS = Post hearing submission, WS = Witness statement, SE = Supplementary exhibits

The contemporary view of case study is that it provides the means to explore complex issues, particularly when human behaviours and social interactions are fundamental to understanding [36]. Case study is also effective for investigating related processes and identifying contextual factors [37]. The case data we investigated had several features worthy of note. Our case data vary in range and depth and the range of perspectives contributing (for example, family members or health professionals). While each of our cases reflects the story of an individual, accounts are provided by other people as such the first-person perspective is absent. Aligned with Merriam’s pragmatist constructionist approach [29] we aimed to reveal the antecedents and processes involved in the chemical restraint of older people receiving residential care. Through cross-case analysis [38], we first categorised and ordered the data across 62 descriptive codes. From there, we connected the subject matter and propositions of the descriptive coding into four key patterns to provide explicit descriptions of the events and processes depicted in the cases. With multiple cases, we have triangulated across the data, and for rigour, we have cross-checked with the academic literature throughout our analysis [39].

## Findings

Seven cases of people in residential care who were prescribed and/or administered psychotropic medication were identified from the data corpus. Three of the cases had been the subject of intense scrutiny at one of the Royal Commission hearings focused on restraint, and the remaining were from four separate hearings in different geographic locations identified through the keyword search. No other cases were identified in the data. The later accounts were not as dramatic but served to demonstrate that chemical restraint can happen in response to what may be considered minor transgressions of behaviour. In four cases, psychotropic medication was prescribed without the knowledge or consent of next-of-kin. All cases included pejorative language to describe dementia behaviours, for example, “wanderer,” “aggressive,” “resistive,” or “combative”, with such language adopted by Counsels Assisting in questions during the hearings. Through our analysis, we demonstrate the problematic and contested nature of these terms with the use of “quotation marks” although some terms may be reproduced in direct extracts throughout our analysis.

Across the seven cases, similarities were identified in the precursors and processes that resulted in as well as challenged chemical restraint. We identified four key patterns of action repeated in the data: labelling and limits of tolerance, pushing prescription as a solution, coverups and avoiding consent, and lastly, family’s fight for liberty. In addition to extracts from the data, our analysis presents findings from the academic literature to demonstrate what is already known about the topics and determine where we identified new information. We have found on presenting this work to colleagues that the findings do ‘ring true’ with clinical experience and, in that way, contribute to documenting knowledge that is known but not published. To protect the identity of Royal Commission informants, extracts are referred to by data source only.

### Labelling and limits to tolerance

In all seven cases, the individual’s behaviours were recognised as the precursor which led to the recommendation and/or administration of psychotropic medication as a means of restraint. Antecedents across the cases related to personal characteristics of the case and the care workforce response. Where older people exhibited behaviours that were identified as uncooperative in the context of the scheduled provision of care, such behaviours were labelled “aggressive”. In addition, behaviours such as calling out and walking around the facility or “wandering” were portrayed as particularly problematic, as indicated by the following extracts.

“He was resistant at times to get up in the morning, like many of us, but often that would relate to the nature of his sleep the night before. They did use the word, that he was showing aggression, and we really questioned that. Resistance is a different thing, I think, altogether than aggression.” (hearing transcript)

“I was told he was not going to the dining room when asked, not coming in from the garden when asked or was walking around during the night.” (witness statement)

In several of the cases, family members either did not see the reported behaviours in situ or they did not consider the re-counted actions to be defiant or combative. Some family members reflected that they did not see resident behaviours as unduly problematic, assuming care staff would have the experience and capacity to work with behavioural symptoms of conditions such as dementia.

“The nurse manager reported the behaviour of concern was that of my mother calling out and screaming. I never saw any evidence of this and was actually quite surprised it was an issue as I had observed a number of residents in high care always calling out and screaming day and night.” (witness statement)

“Given that the wandering is part and parcel of the illness of dementia, I assumed that the facility would be best placed to manage any problems that arose from her wandering – this was not the case.” (witness statement)

“They were supposed to be a dementia specific facility. After two weeks the nurse wanted to medicate/sedate him.” (witness statement)

Using language such as “wandering” and “aggressive” to label resident behaviours as problematic was common. Indeed, reports of “wandering” [40], general agitation [10], and verbal agitation [15] have all been associated with the use of psychotropic drugs in residential aged care. It is noteworthy that in some instances, family members did not see resident behaviours in the same light as care workers. People reported surprise at how specific actions, such as walking around, had been problematised. Families shared the expectation that facilities, some of which were recommended for their specialist care, and their workforces would have the expertise to work appropriately “with” older people, particularly older people with dementia who could no longer be cared for at home.

### Pushing prescription as a solution

Once resident behaviours had been identified and labelled problematic, agents of the facilities in these cases put forward the use of prescription medication as an acceptable way of behaviour management, and approval for prescription medications was sought from family members. Across the cases, the most common recommendation was for prescription of risperidone (also Risperdal), an atypical antipsychotic medication endorsed for people with schizophrenia, bipolar disorder, or irritability associated with autistic disorder to improve thinking, mood, and behaviour [41]. As the following extracts indicate, prescription requests were positioned within a care framework, as a way of avoiding potentially harmful behaviours and rationalised as in the resident’s best interest.

“… the wandering was creating a risk of falling … it was in [resident’s name] best interest and for the sake of his safety that risperidone was increased.” (witness statement)

“He told me that my mother’s safety came first and that is why he supported the administration of risperidone to her.” (witness statement)

In many cases, family members reported experiencing pressure from direct care workers and medical professionals to approve prescription medication. In cases where family members denied initial requests for consent, requests were often repeated. In the case presented below, an ultimatum, an action akin to bullying, resulted in much needed respite care being denied.

“[family] We said ‘no’ on numerous occasions … [counsel assisting] and when you say ‘on numerous occasions’ – is that because the facility was encouraging the prescription of risperidone … [family] yes.” (hearing transcript)

“I was then told that either I agree to the locum medicating with whatever he/she chose to give him or I ‘come and take [resident] home’. Therefore, [resident’s] stay was cut short, and I had no choice but to take him home and manage as best I could.” (witness statement)

Amongst the cases, family members reported being sceptical of intentions to administer medication, knowing that the medication would subdue the resident, with one family member referring to the practice as “behavioural euthanasia”. Families suspected that suggestions to medicate were not in the interest of the individual’s wellbeing but rather as a strategy for easier and uncomplicated care provision.

“I asked the nurses, What did you give her and why? and I was basically told. This is for her behaviour, If we do not give it to her, she will be out of control.” (hearing transcript)

“In short, Risperidone was clearly administered at and for the convenience of [facility name], not as a medical treatment” (hearing transcript)

“I also mentioned about his recommendation to sedate dad. [son] believed that such a prescription might result in behavioural euthanasia” (witness statement)

In a pattern similar to that reported in the “limits of tolerance” finding above, family members again cast doubt over workforce and facility capacity. Here, concerns turned to the penchant to default to prescription medication rather than engage in alternate (nonpharmacological) strategies. In many cases, alternate strategies had been espoused to family members as available and the option of first choice for care provision.

“Oxazepam was to be used in treating my mother only when behavioural strategies had not been successful. I never saw any attempt to use behavioural strategies with my mother.” (witness statement)

Oxazepam is a class of benzodiazepine that works by slowing brain activity to induce relaxation and sedation [42]. A noted side effect of Oxazepam is unsteadiness and a tendency to fall [43]. Similarly, risperidone is known to cause sleepiness, agitation, and dizziness, side effects that may make people prone to falls. While risperidone is approved to treat behavioural symptoms of dementia in Australia, it is recommended as a second line-treatment only and to be used after nonpharmacological methods have been extensively trialled [44]. Elsewhere, including in the USA, risperidone is not approved for use with people who have dementia due to safety concerns [45] and that risperidone is often prescribed off-label [46]. From the informants’ accounts medication appeared to be the first treatment option and not the ‘last resort’ they had expected it to be.

Across all cases, family members expressed concern that medications were pushed for the benefit of the workforce rather than the benefit of the resident. Implicit was the perception that the current workforce lacked the skill, time, training, and or compassion to provide appropriate person-centred care and support. The research literature presents several workforce factors which are associated with high psychotropic medication use in residential aged care, including limited knowledge of behavioural and psychological symptoms among workers, low staff/resident ratio, and lack of access to psychologists or psychogeratricians in residential care [17]. Workloads that do not allow time for non-pharmacological care and limited staff education on dementia has also been linked to medication as a default response to behavioural and psychological symptoms of dementia [19]. Negative staff attitudes toward older people with dementia and labelling behaviours as disruptive and challenging have similarly been associated with higher incidents of chemical and/or physical restraint [21]. While the literature accounts for the role of workforce factors, less appears to be known about family perspectives of pressure to prescribe and the experience of contesting to the administration of psychotropic medication.

### Coverups and avoiding consent

Despite withholding consent, prescribing medication to modify resident behaviour often went ahead. In four of the seven cases, prescriptions were administered without the knowledge and consent of the family. Family members voiced concerns over such practices believing that in their role of next-of-kin, or medical power of attorney, medication should not be prescribed without their informed consent. Some family members seemed to have been kept out of the conversation altogether to the extent that they lacked knowledge about psychotropic medications. In contrast, others who previously had medication related conversations found their agreements and assurances had been disregarded. The following extracts also indicate a lack of transparency around the prescription process.

“… when I arrived on the Sunday, the RN [registered nurse] on duty just mentioned to me saying ‘she’s on some new drugs’. I said, ‘what drugs?’ and they said, ‘It’s risperidone’ and I said, ‘what’s that?” (hearing transcript)

“I was given no explanation as to why they had commenced a new medication without discussing it with me … I reminded her [doctor] that she had agreed that ‘nothing new’ in the way of medications would be prescribed … That was our agreement on admission.” (witness statement)

“We became suspicious that he was being given something … my sister and I asked to see the charts, where we did see that risperidone had been dispensed to my dad.” (hearing transcript)

In addition to the practice of prescribing without consent, evidence from the cases suggested a culture of concealment and poor communication between the workforce at all levels and family members. Family members were disappointed and unconvinced that interactions were ethical and truthful.

“And often when I would ask, ‘has he had any extra medication?’ I would be told no, they did not think so, that he had just had a bad night, and he was very tired. But then when I would check closer … I would find that he had, in fact, had extra.” (hearing transcript)

“Over a number of weeks and months, I tried to ascertain why and how my mum had been prescribed risperidone. I pursued the matter with the then director … I never received a satisfactory explanation.” (witness statement)

For one case, where the family had not approved psychotropic medication, a workaround appeared to have been put in place. In this instance, pain medication in the form of a fentanyl patch was prescribed because the resident “could have been in pain” (witness statement) despite the resident being cleared of pain the previous day. An opioid, fentanyl is recommended for cases of severe pain where other medications have been ineffective; side effects can include drowsiness, confusion, and lack of balance [47].

“He was on fentanyl patches. They said it was for pain, but it wasn’t; it was to manage him. They were sedating him … one of the staff said angrily to me – How else am I going to manage him?” (hearing transcript)

This example shows the extent to which members of the workforce will go in order to bring resident behaviour in line with capacity to provide care. Using a strong opioid for a condition that “could” be, is tantamount to using a medication other than for medical treatment. If this is the intention, such action raises particular concern as using medication in this way is something that sits outside of the consent guidelines. The practice of chemical restraint cannot be consented to by the person responsible for the prescription or the appointed guardian [48]. This unique data set has given us some insight into an alarming pattern of considered actions designed to sedate dementia behaviours deemed too difficult to work. Such actions are imposed upon people already considered vulnerable and without the capacity to provide informed consent and against the expressed wishes of family members. Informed consent for medication is governed by State and Territory Laws. When a person cannot give their own consent, a legally defined substitute decision-maker, such as the person responsible, attorney, or guardian, needs to provide consent. Informed consent cannot be provided by staff members, allied health professionals, or the provider on behalf of the person [49–51]. Psychotropic medication has a history of use in the treatment of dementia behaviours within residential aged care in Australia [13, 52] and in other countries [53–55]. Some of this history includes the use of antipsychotics, without consent from the family, next of kin, or those holding power of attorney [7, 19, 51, 56]. Relatives of people living with dementia in Australian residential aged care homes are documented as expressing their frustrations to learn that psychotropics were administered without their knowledge [19]. In some cases, there have been no records of consent for antipsychotics administered to older people, and in some cases, antipsychotic drugs were prescribed despite families refusing to consent [7].

### Family’s fight for liberty

So far, we have documented concerns about how psychotropic medications were pushed onto older people without family approval. Here we present the mitigating factors, namely family involvement that challenged the prescription and administration of chemical restraints. Across the cases, the reasons for not wanting the older person to be prescribed psychotropic medications were that such medication would negatively impact the individual’s quality of life. It was evident through the witness statements that family members wanted their loved ones to preserve their current level of functioning for as long as possible. As already discussed, family members believed that residential care would offer a level of support that could accommodate the dementia behaviours that they could no longer manage at home. As indicated by the first extract, family members were willing to sacrifice some risk to maintain dignity and quality of life.

“Dad was falling quite a bit … but he never broke anything … we told them that we would rather have dad risk falling than have him chemically restrained with medication.” (witness statement)

“I also made it clear to [Dr] that myself and my siblings did not want him medicated as it would adversely affect his quality of life.” (witness statement)

Despite different levels of health literacy around dementia care, families used the strategies and resources at their disposal to advocate for the support and dignity of their loved ones. As the following extracts demonstrate, some families had access to experts from whom they sought guidance and support to block the administration of medication. Other families used their physical presence to supervise and subvert chemical restraint.

“Both my parents worked in the health sector, and one of my uncles is a psychiatrist. We have family friends who are GP’s and specialists and I queried them about the of using a medication such as risperidone.” (witness statement)

“[daughter] has decides that she will try and visit her mother (and ask brothers to also visit) at around the time she receives her medication to monitor for over sedation.” (Exhibit)

Although engaging residential care services for respite or permanent support, this was not a time of rest for family members. In one case of respite care, a family member was repeatedly notified about “problematic” behaviour, in other cases, families had to work hard to ensure that alternative treatments and approaches of care were used in favour of chemical restraint.

“While [resident’s name] was there, I received phone calls from the nurse five to six times per day telling me of things that they [the resident] had done.” (witness statement)

“I worked really hard in conjunction with the facility and with his carers that we devised a plan to minimise the frustration that he had … but that took a lot of work.” (witness statement)

Placing a family member in residential care is a complex decision [57–59]. In instances of cognitive impairment, families take on the caring role until they can no longer do so [59, 60]. When the move to residential care is made, even for respite purposes, it is reasonable to expect that the host facility can meet individual’s needs and that the role of the family in care provision can be reduced. In the examples provided here, this has not been the case. Family members have had to work hard to fight for the rights of their loved ones as well as consistently monitor residential care delivery. Family members with neither the clinical knowledge nor appropriate supports have spoken out to challenge the intentions of care support workers and health practitioners.

While family involvement following the placement of a relative with dementia into care has been shown to help improve family staff relationships and communication and improve the quality of life for the resident [61], the type of vigilance and advocacy that is evidenced here goes beyond what family members had anticipated. The expectation of being able to rely on the knowledge, skills, and capacity of the care support workforce to be able to provide care that supports quality of life has not been met. Indeed, previous Australian research has shown that lack of staff responsiveness is the reason why families need to intervene and advocate for the rights of the person receiving care [62]. This raises the question, what happens to older people who do not have family or friends with the knowledge and resources to advocate for their needs?

## Discussion

The Royal Commission reports (both interim and final) provided frequent general references to the use, misuse, and general lack of monitoring or regulation for restraints and for chemical restraint use. While the Royal Commission made recommendations for better dementia care the term “chemical restraint” was used just once within the 148 recommendations, and it was in the context of *recommending* use “only if prescribed by a doctor who has documented the purpose of the prescription” [63]. As our findings have demonstrated, any level of endorsement may be all that is needed for chemical restraint to be used as a “first resort” option for care. This study aimed to analyse the Royal Commission evidence to describe circumstances that the public identified as influencing psychotropic prescriptions for older people in residential aged care. To do this, we have explored the data of seven individuals whose families, we can presume in the hope of making a difference, submitted their stories of unacceptable care to the Royal Commission.

Caring for older people with dementia can be challenging for informal and formal carers alike [64–66]. Where families can no longer provide care and support in the home, alternate residential care can be necessary, on a permanent or respite basis. Our findings indicate that the families represented in our cases have enlisted residential care with the belief and expectation that professional and specialist care would be provided at a level that met the needs of their family members. In all cases, expectations of care and support have not been met. In addition, due to the constant vigilance required to ensure care, families seeking respite had no reprieve, and those seeking a more permanent care solution remained physically present to ensure appropriate care, consequences which were neither anticipated nor practical. As mentioned, the families reflected in our cases, to varying degrees, remained highly active in their loved one’s care following transfer to residential care. However, such familial support may not be available to everyone, nor we argue, should it be required to ensure appropriate dementia care and support are delivered. With close to 40% of people in residential care in Australia not having visitors [67] we cannot rely on the presence of family and friends to monitor care. It is important that trust is re-established and for appropriate person-centred dementia care and support to be delivered regardless of external presence and advocacy.

Decrease in the use of physical restraints in residential aged care has been linked to the increasing use of chemical restraints [68] and our findings are a further indication of this trend. Any discussion of chemical restraint and informed consent must acknowledge the inherent paradox. As mentioned, using a medication for a purpose other than medical treatment, for example to modify behaviour, is something that cannot be consented to [48]. Therefore, it stands that informed consent can neither legitimately be sought nor provided for chemical restraint. This may be why, in more than half of our cases medications were prescribed or administered without the knowledge of family members. This may also explain the extent to which “work arounds” such as pain medication were used to effect behaviour modification. We acknowledge that there are legitimate circumstances for psychotropic medications to be used in the treatment of dementia behaviours. However, such prescription needs to be considered a last resort and involve consultation with experienced specialists such as psycho-geriatricians [5]. There was no mention of such a consultation process in any of our cases. Also absent from our evidence is any attempt to consult with the person living with dementia by clinical staff or families. While there may be fluctuation in decision making capacity, it should not be assumed that people living with dementia cannot make or contribute to decisions about their own medical treatment, including decisions around medications [5].

We are neither the first to use evidence from this Royal Commission for scholarly analysis [69, 70], nor are we the first to investigate the topic of dementia behaviours and restraint [71]. However, to our knowledge, we are the first to systematically search all the hearings and exhibits and perform in-depth qualitative analysis. Despite limited prior examples of how to analyse large qualitative data sets our comprehensive search strategy and methodical approach to data extraction gives us confidence that we have captured and analysed the data most relevant to our aim. In addition, our explicit consideration for the ethical implication of using this data has not previously been contemplated [69, 71] and is also to date, unique to our methodological and analytical approach [72]. Our analysis has taken seven stories and applied a scholarly lens to the data while at the same time respecting the privacy of the informants and the sensitive nature of all their accounts. In line with our pragmatist constructionist approach, we have added to what is known about the precursors and practices of chemical restrain in Australian residential aged care.

This secondary analysis aimed to make use of an unprecedented collection of accounts of aged care in Australia. However, we must acknowledge that data of this scale and type has inherent limitations and constraints. Our pragmatist approach to the data meant we focused our attention on cases. While we were able to produce an in-depth analysis, and are confident that we captured vital content, we do not claim to have captured everything that could be known from the data corpus. Our analysis is inherently limited to the evidence presented to the commission and the investigative direction of the Council Assisting. Our cases also varied in their volume and sources. We could not ask questions of the informants or delve further into any issues. The evidence presented to the Royal Commission has been generated by a specific ‘data-public’ [73] a group of people with a vested interest in a topic. In this instance, the data comes from people who have had previous negative experiences in the aged care system, and who care enough to submit their stories to a public forum, as such our data comes with an inherent bias. We know that best practice in health and social research includes the voice of the persons impacted [74, 75]. For a typical qualitative investigation on dementia and restraint, such investigation would ideally involve the voice of the older person. The voices included in our analysis are those that tell a story *about* an older person, they are not first-person accounts. While this is not an intentional exclusion, we acknowledge that our conclusions and recommendations are constrained by the voices contained.

Eliminating the use of chemical restraint in residential aged care will be a complex undertaking, requiring change on multiple levels and from many people. Increasing workforce capacity in terms of number and skill set has been a central recommendation from the Royal Commission [62]. However, more is needed to support older people in residential care and their families. Regarding workforce education, staff empathy and knowledge of restraint regulations and the dangers of restraint use are essential starting points [19]. Raising the public’s awareness and educating family and friends will be more complex. Our evidence shows that there are family members who can recognise the signs and symptoms of chemical restraint. However, relying on family to be able to observe the impact of medication is not a solution. It is also clear that there is work to be done on the language used to describe dementia behaviours. As noted, the Commissioners and the Counsel Assisting adopted the terms “wandering” “aggressive” and “challenging” when summarising response. The use of such language not only dehumanises the individual but it also pathologises behaviours typically associated with dementia and reinforces the need for a pharmacological response [71]. Changing the way dementia responses and behaviours are perceived both medically and publicly will be imperative for changing the way people with dementia are supported in residential care as well as in their homes and communities.

## Data Availability

All data used for this project is publicly available from https://agedcare.royalcommission.gov.au/.

## Declarations

### Twitter

Centre for Research in Aged Care @crac_ecu.
